# Responses of Urban Bird Assemblages to Land-Sparing and Land-Sharing Development Styles in Two Argentinian Cities

**DOI:** 10.3390/ani13050894

**Published:** 2023-03-01

**Authors:** Maximiliano A. Cristaldi, Ianina N. Godoy, Lucas M. Leveau

**Affiliations:** Departamento de Ecología, Genética y Evolución, Facultad de Ciencias Exactas y Naturales, Universidad de Buenos Aires-IEGEBA (CONICET-UBA), Ciudad Universitaria, Pab 2, Piso 4, Buenos Aires 1426, Argentina

**Keywords:** avian, Latin America, species composition, taxonomic diversity, urban planning

## Abstract

**Simple Summary:**

Urbanization negatively affects biodiversity worldwide. As cities are expected to grow in the future, alternative urban developments which allow the conservation of biodiversity within cities are required. Our main aim was to compare the response of bird assemblages to two alternative urban development styles (land-sparing vs. land-sharing) in two Argentinian cities: Santa Fe and Buenos Aires. Additionally, we assessed the response of bird assemblages to landscape features (the coverage of vegetation and distance to the main rivers) and human activity (represented by pedestrian rate and environmental noise). In Buenos Aires, land-sparing enhanced species richness, whereas land-sharing favored the Shannon diversity and Simpson diversity. The pedestrian traffic was negatively associated with bird diversity. We found that each urban development style supported different bird assemblages during the breeding season. Bird species composition was also related to the surrounding coverage of vegetation. Therefore, our study shows that both urban development styles support different bird assemblages, especially during the breeding season, and indicate the need of reducing pedestrian traffic and increasing the coverage of vegetation to enhance species diversity and composition in both cities.

**Abstract:**

Urbanization negatively affects biodiversity worldwide. Consequently, alternative urban development styles are required for an eco-friendlier urbanization process. Thus, two development styles have been suggested: land-sharing (buildings mixed with dispersed green space) and land-sparing (buildings interspersed with large green patches). We assessed differences in species diversity and composition of bird assemblages between both development styles in two Argentinian cities: Santa Fe and Buenos Aires. We surveyed birds in land-sharing and land-sparing areas during the breeding and non-breeding seasons. As a control, we also surveyed birds in areas dominated by impervious surfaces. At a local scale, we also measured the environmental noise and pedestrian traffic. At a landscape scale, we measured the percent vegetation cover surrounding development styles and their distance to the main river. In Buenos Aires, species richness was higher in land-sparing than in land-sharing. However, the Shannon diversity and Simpson diversity were higher in land-sharing. In Santa Fe, both urban development styles supported similar species richness and diversity. Species composition varied between land-sharing and land-sparing in both cities during the breeding season. The pedestrian traffic was negatively associated with species diversity. Therefore, both development styles and strategies to reduce pedestrian traffic should be taken into account to enhance different components of species diversity and composition within the urban matrix.

## 1. Introduction

Urbanization is a complex socioeconomic process that has grown globally in recent decades [1]. The world urban population increased by about 1.973 million inhabitants and the global urban surface area grew by about 80% between 1985 and 2015 [1,2]. This accelerated urban development is leading to a strong transformation of the landscape and natural ecosystems [3,4]. Numerous studies at a global scale have indicated that urbanization impacts biological communities causing changes in their ecological interactions, the behavior of individuals, and their biodiversity [5,6,7]. Intensive urban development leads to the loss of species, mainly native species [7,8]. Due to the negative impacts of urbanization on natural ecosystems, it is necessary to seek an alternative urban landscape planning that allows for biodiversity conservation [9]. 

A multidisciplinary debate was recently raised between two models of urban development: land-sharing vs. land-sparing [10,11]. The land-sharing model refers to the expansion of low-intensity urban areas that contain relatively small green spaces in a dispersed manner [11]. In contrast, the land-sparing model promotes urban densification in some sectors to the extent that relatively large green spaces are established [11]. Several studies have discussed the relevance of each model in different cities and taxonomic groups such as vegetation, beetles, mammals, and birds [11,12,13,14]. Particularly for birds, the most current understanding of the response of bird assemblages to land-sparing and land-sharing development styles in urban environments is spatially biased towards a few cities [15,16] or regions [14] and with contrasting results. Additionally, urban landscapes are spatially and temporally heterogeneous, and consequently, the response of biological communities to land-sharing/land-sparing models may depend on factors such as the geographical, biological, and social context [13,14]. The extent to which the patterns are generalizable remains unclear. Therefore, this highlights the importance of conducting studies in different cities to regionally differentiate the suitability of each urban development style for bird diversity [17]. 

In urban areas, birds are one of the most frequently studied taxa, since they rapidly respond to anthropogenic changes, are easy to survey, and can function as surrogates of diversity for other taxa [18]. Birds also provide ecosystem services such as pollination, seed dispersal, and pest control, and bird species richness may have a positive impact on human well-being [19,20,21,22]. Although in recent years the study of urban birds has grown rapidly in highly biodiverse regions of Latin America such as Brazil, Argentina, and Mexico [23], gaps in the knowledge of the effect of urbanization on bird assemblages still remain [23,24]. Additionally, as in many works from temperate zones (i.e., North America, Europe, and Australia) [25], studies from Latin America have reported a negative effect of urbanization and a positive influence of green space size on bird diversity [26,27,28,29,30]. Since this region is one of the most diverse in avian species, but at the same time it is experiencing an unplanned lower-density growth in many cites [23], knowledge about the effect of urban development styles on urban bird assemblages is imperative as a base to guide future eco-friendlier urbanization.

The effect of urbanization on bird diversity constitutes a process that varies according to different spatial scales [17,31]. At the local scale, an increase in bird diversity is usually associated with greater plant diversity and stratification, the presence of natural or artificial waterbodies, and low levels of human disturbance such as pedestrian traffic and noise [31,32]. At the landscape scale, greater species richness has been associated with greater proximity to waterbodies or green spaces in different cities [33]. However, the relation between bird diversity and land-sharing/land-sparing models has not been analyzed considering the distance to other waterbodies or green areas.

Moreover, the relationship between bird diversity and urbanization may change between seasons [34,35]. In general, birds have a more restricted home range during the breeding season since they are associated to a nesting territory, whereas during the non-breeding season, birds have a more flexible home range because they are mainly influenced by the abundance and distribution of food [36,37]. Therefore, the relationship between bird diversity and land-sharing/land-sparing models needs to be analyzed in different seasons [14].

In this study, our aims were (1) to compare bird communities in land-sharing and land-sparing landscapes; (2) to analyze the role of human disturbance represented by pedestrian traffic and level of noise on bird communities; (3) to analyze the role of the amount of green cover and the distance to the main watercourses of urban landscapes on bird communities; and (4) to compare the relation between bird communities and different landscapes during breeding and non-breeding seasons. We expected higher bird diversity in land-sharing during the breeding season due to the presence and higher abundance of species that can nest on manmade structures and use the surrounding natural resources. In addition, we expected higher bird diversity in landscapes surrounded by more green cover and next to watercourses. Finally, we expected differences in bird diversity and species composition related to environmental variables only during the breeding season.

## 2. Materials and Methods

### 2.1. Study Area and Preliminary Classification of Urban Areas

**Figure 1:**
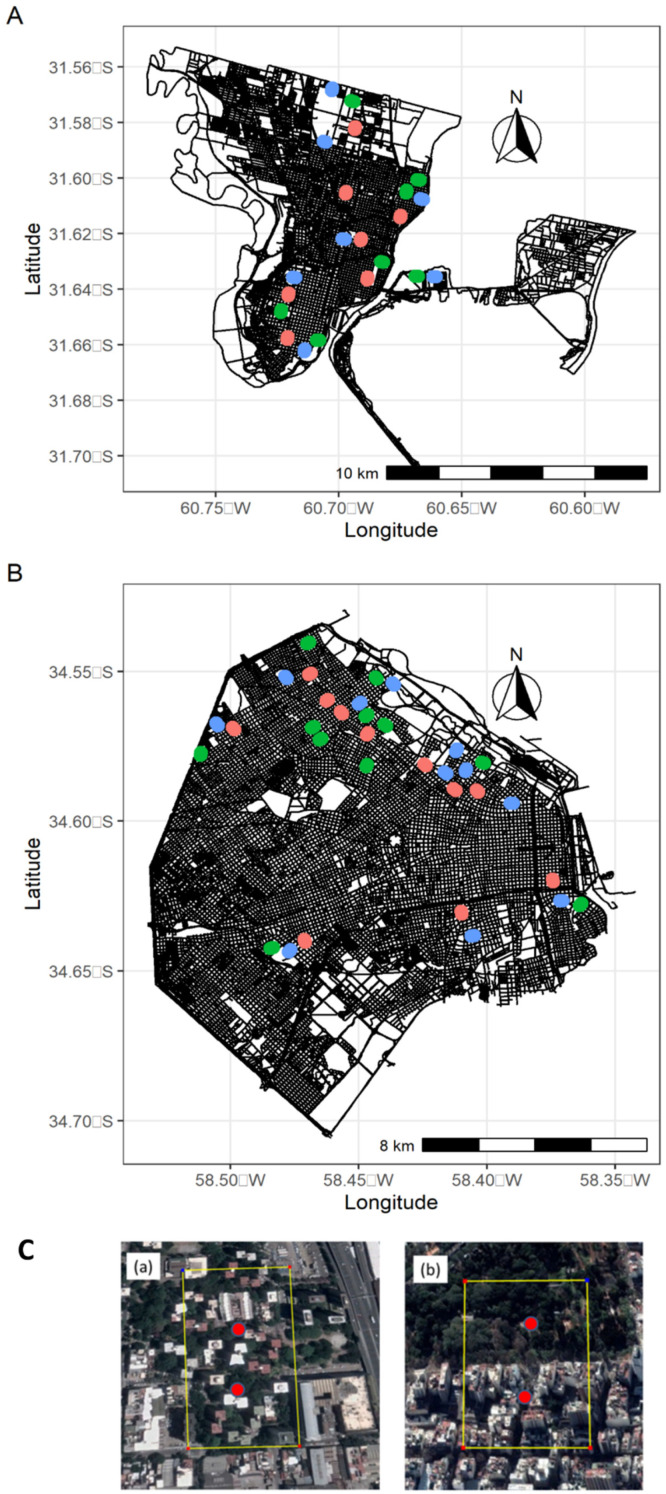
Location of control (red circles), land-sharing (green), and land-sparing (blue) landscapes in Santa Fe (**A**) and Buenos Aires (**B**) city. Examples of sample units used in this study: (**C**(**a**)) land-sharing and (**C**(**b**)) land-sparing landscapes. The yellow line indicates the limits of the sample unit (300 × 200 m), whereas red points indicate the location of point counts.

We assessed the taxonomic diversity and composition of bird assemblages in two cities from central Argentina, Buenos Aires (34°35′59″ S 58°22′55″ W, 3,075,646 inhabitants, 25 masl) and Santa Fe (31°38′00″ S 60°42′00″ W, 401,544 inhabitants, 25 masl) (Figure 1A,B). Buenos Aires is located in an ecotone between the Pampean and the Paranaense phytogeographic regions [38]. The Pampean region was originally dominated by grasslands, whereas the Paranaense region is composed of deltaic forests. Santa Fe is located in the ecotone between the wooded Espinal and the Paranaense phytogeographic regions. However, the surroundings of both cities are heavily impacted by human activities, being dominated by crops and exotic tree plantations.

In each city, we selected 200 m × 300 m sampling units depending on the availability of landscapes with land-sharing or land-sparing (14 in Santa Fe city and 22 in Buenos Aires city) (Figure 1A,B). Half of them presented a land-sharing urban development and the other half, a land-sparing urban development (Figure 1A–C). To recommend a land-sharing or land-sparing urban development style for future urban planning, we need to provide evidence that at least one of them supports a different bird assemblage from highly urbanized areas. Thus, we considered 200 m × 300 m rectangles where pavement and buildings predominated (>50% impervious cover) and hereafter referred to them as “control” (7 in Santa Fe city and 11 in Buenos Aires city). Sampling units were separated by a minimum distance of 200 m to secure data independence. Sampling units were initially assigned to either one or another urban development style by visual inspection of satellite images available on Google Earth. Land-sharing units consisted of fragmented green areas interspersed by buildings, while land-sparing units corresponded with the majority (>50%) of their green surfaces aggregated into a single patch (Figure 1C). All green spaces presented a certain level of management or human intervention, such as lawn mowing, irrigation, or pruning. Due to a positive relationship between the size of green areas and animal biodiversity (including birds) that has been extensively reported in urban landscapes [29,39], every land-sharing square in a given city was paired with another land-sparing square of the same city holding a similar overall green area. This procedure allowed us to test for the effect of urban landscape organization, avoiding bias associated with the size of green areas. 

### 2.2. Classification of Urban Areas

In order to confirm the initial assignment of each sampling unit to one of three urban development styles, we followed Ibáñez-Álamo et al. [14]. Using the satellite images from Google Earth and ImageJ package [40], we divided each 200 m × 300 m rectangle into 96 cells (25 m × 25 m) and estimated the percentage of vegetated and non-vegetated surface for each cell (Figure S1). Then, we used this information to calculate the following variables for each rectangle: (Single_patch), percentage of high vegetation cover cells (those with more than 50% green area) in a single patch (contiguous cells); (N_patches), number of green patches (a green patch was defined as having at least one high vegetation cover cell); (Per_built_cells), percentage of built cells of all vegetated cells; (Per_veg_cells) percentage of fully vegetated cells of all vegetated cells; (N_veg_cells), number of cells with vegetated surfaces; and (Per_veg_cover), percentage of vegetation cover in the sampling unit. Variables Single_patch and N_patches provide information on the land-sharing/sparing urban development style at the 200 m × 300 m square level with high values of variable Single_patch associated with land-sparing urban areas (i.e., vegetation in a single patch), while high values of variable N_patches are associated with land-sharing urban areas (i.e., vegetation distributed into many patches). Variables Per_built_cells and Per_veg_cells provide information on the within-cell land-sharing or land-sparing urban development, respectively, and variables N_veg_cells and Per_veg_cover estimate the overall amount of vegetation in the square [14]. Since the width of greenways is often positively associated to the flow of individuals of many urban bird species [41,42,43], we used the Rook contiguity criteria to determine contiguous cells. With the previous six variables, we ran a principal component analysis (PCA) using the FactoMineR package version 2.7 [44] from R [45]. The first two PCA axes retained more than 80% of data variation for both cities (Figure S2A,B). For both cities, the first axis contrasted control, land-sparing, and land-sharing rectangles based on variables: Single_patch, Per_veg_cover, Per_built_cells, and Per_veg_cells. The second axes contrasted land-sharing from land-sparing and control rectangles based on N_patches and N_veg_cells (Figure S2A,B). Thus, we confirmed the suitability of our initial classification (Figure S2).

### 2.3. Bird Survey

Data on bird species were collected using fixed-radius point counts [46] carried out during the 2020 non-breeding season (May–August) and breeding season (October–December). Standardized point count surveys have been recommended to provide data resulting in indices of abundance that are comparable across years, habitats, and studies [47]. In urban environments, point counts are as effective as other widely used techniques in determining patterns of relative abundance [48]. Two professional ornithologists from Santa Fe and Buenos Aires with more than 10 years of bird-survey experience in their respective regions carried out all the surveys in each city. Within each 200 m × 300 m rectangle, 2 point counts were settled with a minimum of 100 m distance between them and from the border of the rectangle to avoid counting the same individual twice (Figure 1C). At each point count, we recorded all birds seen and heard for 5 min within a 30 m radius. We considered all individuals perching, nesting, or feeding within the point counts for further analyses. Point counts were carried out during the morning (up to 4 h after local sunrise), only in working days to avoid excessive variation in the circulation of vehicles and people and with similar weather conditions (without rain and heavy winds). To capture potential temporal changes in bird assemblages within season, we carried out three surveys separated by a month in each season. 

### 2.4. Environmental Variables

Environmental variables were established at two different scales. At a landscape scale, we analyzed the distance to the nearest river and the vegetation coverage surrounding the sampling units. Landscape variables were estimated using a global land cover map with 10 m pixel resolution [49]. In order to measure the distance to the nearest river, we calculated the Euclidean distance between the centroid of each sampling unit and the nearest river using the raster package version 3.6-11 [50] (Hijmans, 2022). In order to calculate the vegetation coverage, we performed the following steps. First, the original 23 land use types were reclassified into 2 broad categories: vegetated and non-vegetated areas. Second, we made buffers of 500 m width from sampling units using the rgeos package version 0.6-1 [51]. Finally, we calculated the vegetation coverage using the landscapemetrics package version 0 [52].

At the sampling unit scale, we measured the pedestrian traffic and the environmental noise. To measure the pedestrian traffic, we recorded the number of people passing through the point count surface during bird surveys. Environmental noise was measured using the cellphone application “Sound Meter” [53] as per de Camargo Barbosa et al. [31]. The mean decibels per 30 s immediately before and after bird counts were estimated. Due to the fact that the sound meter was not calibrated, decibels measures should be taken only for relative comparisons between urban development styles.

### 2.5. Data Analysis

#### 2.5.1. Taxonomic Diversity per Urban Development Style 

For each season, we measured taxonomic bird diversity using Hill numbers, which are the effective numbers of equally abundant species [54]. Hill numbers differ by a parameter *q* that reflects their respective sensitivity to the relative frequency of a species. We used the hillR package version 0.5.1 [55] to calculate Hill numbers with q = 0, q = 1, and q = 2, which can be interpreted as bird species richness (BSR), Shannon–Wiener (H), and Simpson’s (S) index of diversity, respectively [56]. Bird species richness was the total number of species, whereas Shannon–Wiener and Simpson diversities reflected the number of common and dominant species, respectively [56]. To ensure our survey effort was comparable between urban development styles, we calculated rarefaction curves for each urban development style and city in relation to sample completeness using 999 bootstraps [57] with the iNEXT package version 3.0.0 in R [58]. Sample completeness is the proportion of the total individuals that belong to the species detected in the sampling unit [58]. Significant differences between curves were established when 95% confidence intervals did not overlap (Figure S3).

#### 2.5.2. Taxonomic Diversity per Sampling Unit

BSR was calculated as the maximum number of recorded bird species at each sampling unit considering the three surveys within each season, whereas Hill numbers for Shannon–Wiener and Simpson diversities were calculated with the maximum individuals for each species recorded during the three visits. Environmental variables included urban development styles, cities, pedestrian traffic, noise, and landscape variables. The interactions between urban development styles and city and between urban development styles and landscape vegetation cover also were explored. Generalized linear models (GLMs) were performed to analyze association patterns between bird diversity (Hill numbers) and environmental variables. Models were obtained by backward elimination of non-significant variables (*p* > 0.05) from the full model using the anova function. Final models were compared with null models using a likelihood ratio test (LRT test) (*p* < 0.05). Differences of means between types of urban development styles were explored with Tukey tests using the function glht of the multcomp package version 1.4.20 [59]. Multicollinearity among predictor variables was explored using the vif function of the car package version 3.1.1 [60]. As gvif values were lower than 5, all variables were retained for further analyses. The pseudo-rsquare of final models were obtained using piecewiseSEM package version 2.1.2 [61]. The final models were plotted with the visreg package version 2.7.0 [62]. For species richness (q = 0) (count data), we assumed a Poisson distribution of errors and we checked for over- and sub-dispersion. For the Shannon–Wiener and Simpson index (q = 1 and q = 2, respectively) (continuous data), we assumed a Gaussian distribution of errors, and homoscedasticity and normality were checked. All diagnostic analyses were carried out with the DHARMa package version 0.4.6 [63] (see Figure S4 for model diagnostics).

#### 2.5.3. Taxonomic Composition

To estimate the variation in species composition explained by environmental variables in each season, we used a distance-based redundancy analysis (dbRDA) using the vegan package version 2.6.2 [64,65]. db-RDA is an ordination method which arranges data objects in a space defined by the linear combinations of explanatory (environmental) variables and, at the same time, quantifies the variation in species composition explained by the environmental variables [64,65]. dbRDA is a reliable test for analyzing species–environment relations, especially with linear environmental gradients [66]. The variation of species composition in db-RDA has to be expressed on the basis of a non-Euclidean distance response matrix. We examined a Bray–Curtis dissimilarity matrix along with urban development styles and environmental variables. Environmental variables included urban development style, cities, pedestrian traffic, noise, and landscape variables. Models were obtained by backward variable selection and comparisons with null models using a likelihood ratio test (LRT test) (*p* < 0.05). A db-RDA with the variable “Urban development style” as significant makes it possible to determine that species composition is different in at least one of the urban styles. However, our main objective was to determine the variation in bird species composition between land-sharing and land-sparing. Thus, if db-RDA showed significant differences between development styles, we would perform a new db-RDA considering only land-sharing and land-sparing (hereafter, “db-RDA2”). 

## 3. Results

In Buenos Aires city, we recorded a total of 6001 individuals of 48 species (Table 1). The most abundant species were *Columba livia* and *Zenaida auriculata*. In Santa Fe city, we recorded a total of 4391 individuals of 63 species (Table 1). The most abundant species were *Zenaida auriculata* and *Passer domesticus*. 

### 3.1. Taxonomic Diversity per Urban Development Style

**Figure 2:**
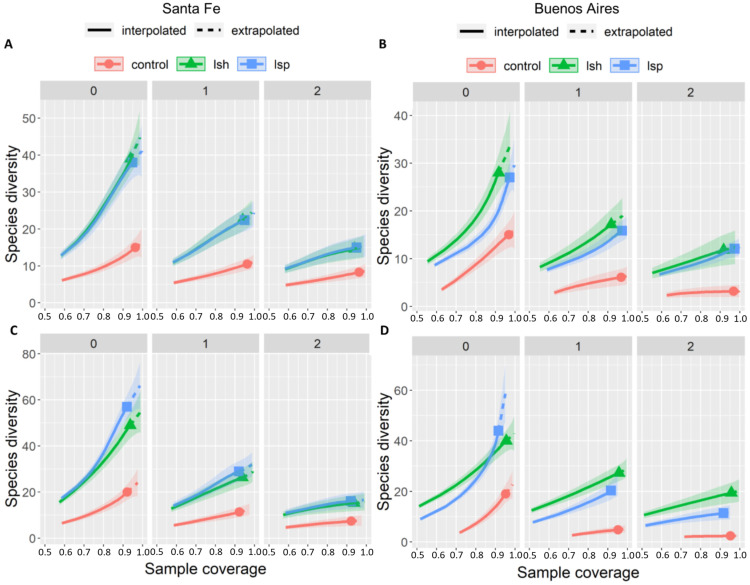
Rarefaction curves of Hill numbers (species richness, q = 0; Shannon diversity, q = 1; and Simpson diversity, q = 2) in relation to sample coverage for control, land-sharing (lsh), and land-sparing (lsp) landscapes during the non-breeding (above) and breeding seasons (below) in Santa Fe and Buenos Aires city (**A**–**D**, respectively), Argentina. Shaded bands indicate 95% confidence intervals.

During both seasons, species diversity in control was lower than in land-sharing and land-sparing for all Hill numbers (Figure 2A–D). In Santa Fe city, species diversity did not differ between land-sparing and land-sharing during both seasons (Figure 2A,C). Although we observed a higher species richness and Shannon diversity in land-sparing than in land-sharing during the breeding season, we did not find significant differences (Figure 2C). 

In Buenos Aires city, species diversity did not differ between land-sparing and land-sharing during the non-breeding season (Figure 2B). During the breeding season, species richness was higher in land-sparing than in land-sharing, whereas Simpson diversity was higher in land-sharing than in land-sparing (Figure 2D). Shannon diversity did not differ between these two landscapes during both seasons (Figure 2B,D). 

### 3.2. Taxonomic Diversity per Sampling Unit

**Figure 3:**
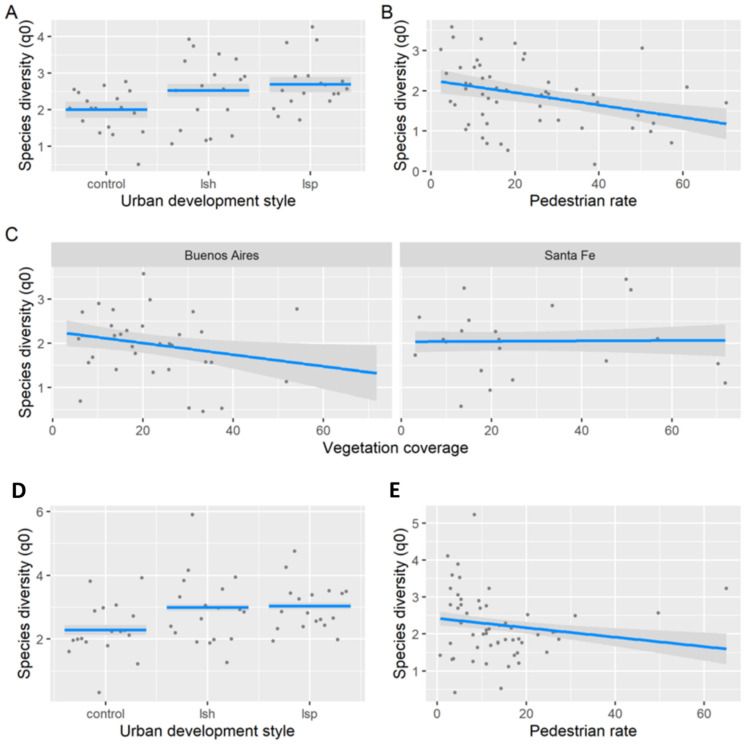
Representation of the best generalized linear model for species richness (q = 0) in Buenos Aires and Santa Fe (Argentina). Species richness in relation to urban development style (**A**), pedestrian rate (**B**), and the interaction between the percentage of surrounding vegetation coverage and city (**C**) during the non-breeding season. Species richness in relation to urban development style (**D**) and pedestrian rate (**E**) during the breeding season. Abbreviations: lsh—land-sharing; lsp—land-sparing. Grey dots refer to sampling units (200 m × 300 m rectangles). Blue lines indicate the parameter estimates with 95% confidence intervals represented by the grey area.

Taxonomic diversity responded differently to environmental predictors at both landscape and local scales between seasons. During the non-breeding season, species richness was related to urban development styles, pedestrian rate, and the percentage of surrounding vegetation coverage (LRT = 79.69, df = 6, *p* < 0.001, pseudo-R^2^ = 0.77). Species richness was lower in control than in land-sparing and land-sharing, whereas we did not find significant differences between land-sparing and land-sharing (Tukey test, *p* > 0.05) (Figure 3A). Pedestrian traffic was negatively associated with species richness (Figure 3B). The relationships between species richness and surrounding vegetation coverage varied between cities (Figure 3C). In Buenos Aires, species richness related negatively to vegetation coverage, whereas in Santa Fe, there was no clear relationship between variables (Figure 3C). During the breeding season, we also found lower species richness in control landscapes and negative relationships with pedestrian rate (LRT = 110.26, df = 3, *p* < 0.001, pseudo-R^2^= 0.87, Table 2, Figure 3D,E).

**Figure 4:**
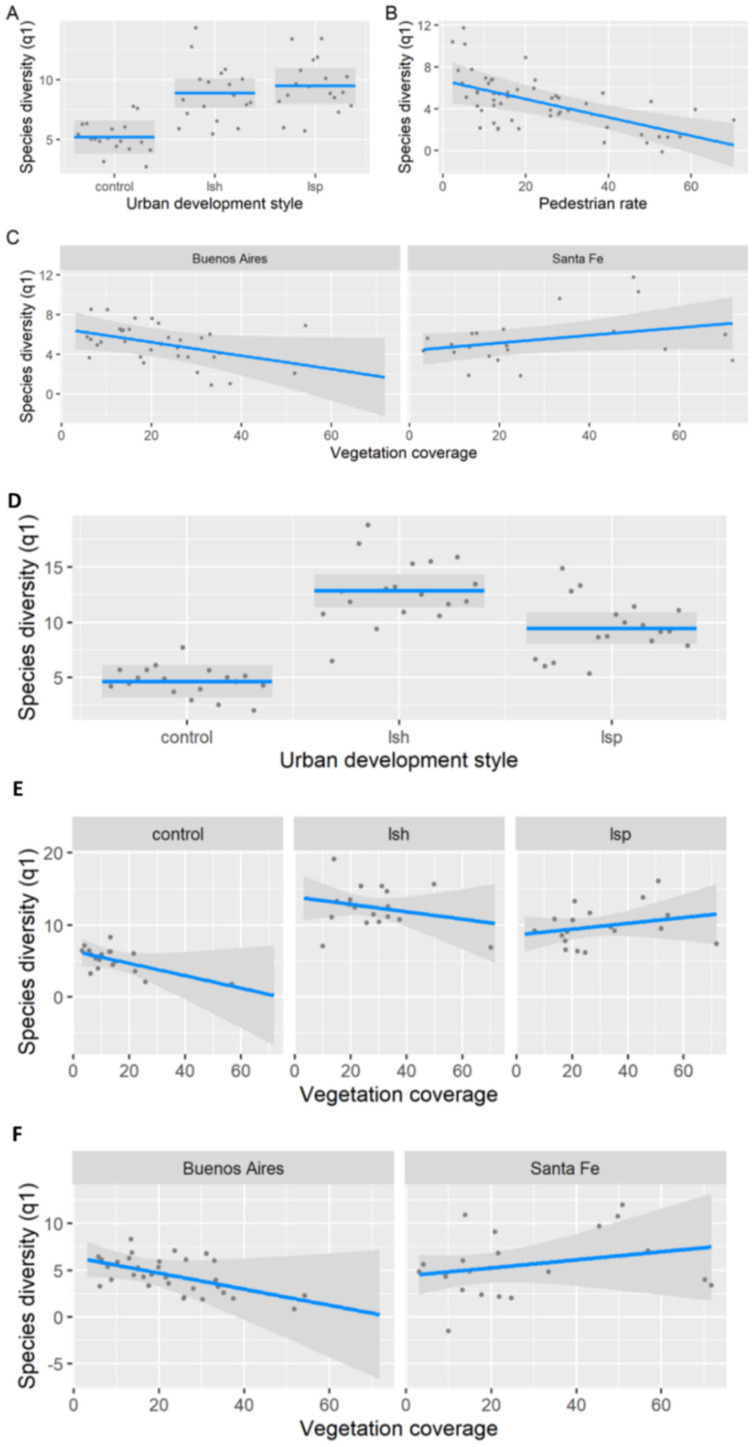
Representation of the best generalized linear model for Shannon diversity (q = 1) in Buenos Aires and Santa Fe (Argentina). Shannon diversity in relation to urban development style (**A**), pedestrian rate (**B**), and the interaction between the percentage of surrounding vegetation coverage and city (**C**) during the non-breeding season. Shannon diversity in relation to urban development style (**D**), the interaction between the urban development style and the percentage of surrounding vegetation coverage (**E**), and the interaction between the surrounding vegetation coverage and city (**F**) during the breeding season. Abbreviations: lsh—land-sharing; lsp—land-sparing. Grey dots refer to sampling units (200 m × 300 m rectangles). Grey dots refer to sampling units (200 m × 300 m rectangles). Blue lines indicate the parameter estimates with 95% confidence intervals represented by the grey area.

During the non-breeding season, Shannon diversity was related to urban development styles, pedestrian rate, and the percentage of surrounding vegetation coverage (LRT = 367.73, df = 6, *p* < 0.001, pseudo-R^2^ = 0.63; Table 2; Figure 4A–C). Shannon diversity was lower in control than in land-sparing and land-sharing (Tukey tests, *p* < 0.05; Figure 4A). We did not find significant differences between land-sparing and land-sharing (Tukey test, *p* > 0.05). Pedestrian traffic was negatively associated with Shannon diversity (Figure 4B). The association between the surrounding vegetation coverage and Shannon diversity varied between cities, being negative in Buenos Aires and positive in Santa Fe (Figure 4C). During the breeding season, Shannon diversity was related to urban development styles and the percentage of vegetation coverage (LRT = 744.4, df = 7, *p* < 0.001, pseudo-R^2^ = 0.71; Table 2; Figure 4D–F). Shannon diversity was higher in land-sharing than in land-sparing and control (Tukey tests, *p* < 0.05; Figure 4D). The association between Shannon diversity and surrounding vegetation coverage varied between urban development styles and cities (Figure 4E,F). Control and land-sharing had a negative relationship, whereas land-sparing had a positive relationship (Figure 4E). On the other hand, the relationship between Shannon diversity and vegetation cover was negative in Buenos Aires and positive in Santa Fe (Figure 4F). 

**Figure 5:**
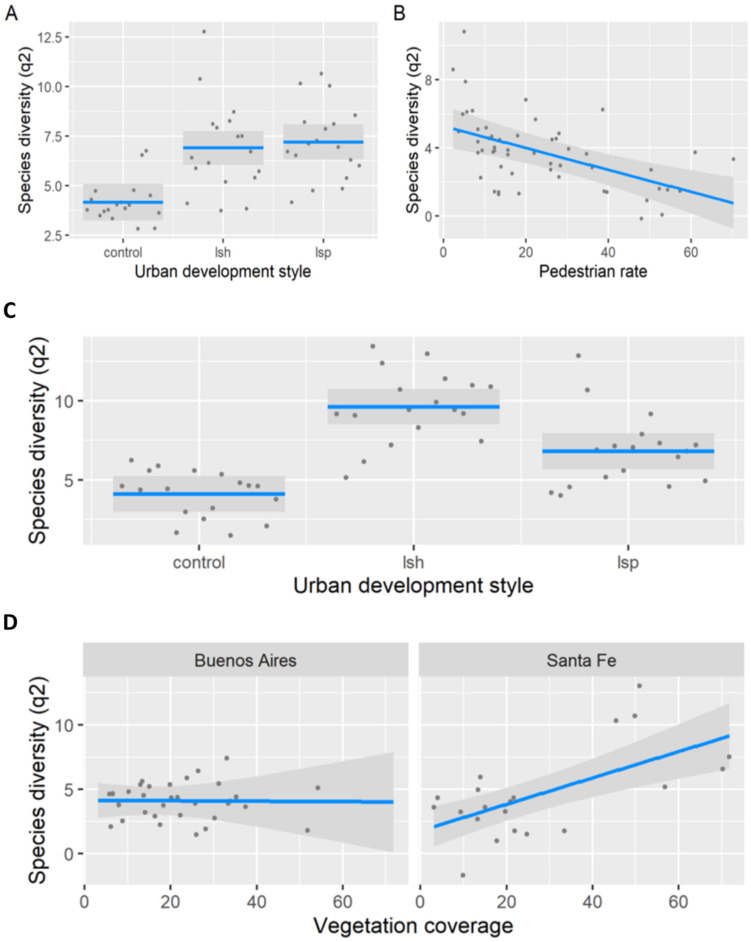
Representation of the best generalized linear model for Simpson diversity (q = 2) in Buenos Aires and Santa Fe (Argentina). Simpson diversity in relation to urban development style (**A**) and pedestrian rate (**B**) during the non-breeding season. Simpson diversity in relation to urban development style (**C**) and the interaction between the percentage of surrounding vegetation coverage and city (**D**). Abbreviations: lsh—land-sharing; lsp—land-sparing. Blue lines indicate the parameter estimates with 95% confidence intervals represented by the grey area.

During the non-breeding season, Simpson diversity was related to urban development styles and pedestrian rate (LRT = 197.39, df = 3, *p* < 0.001, pseudo-R^2^ = 0.53; Table 2; Figure 5A,B). Simpson diversity was lower in control than in land-sparing and land-sharing (Tukey tests, *p* < 0.05; Figure 5A). We did not find significant differences between land-sparing and land-sharing (Tukey test, *p* > 0.05). Pedestrian traffic was negatively associated to Simpson diversity (Figure 5B). During the breeding season, Simpson diversity was related to urban development styles and the percentage of surrounding vegetation coverage (LRT = 419.93, df = 5, *p* < 0.001, pseudo-R^2^ = 0.66; Table 2; Figure 5C,D). Simpson diversity was higher in land-sharing than in land-sparing and control (Tukey tests, *p* < 0.05; Figure 5C). The association between Simpson diversity and surrounding vegetation coverage was negative in Buenos Aires city and positive in Santa Fe city (Figure 5D). 

### 3.3. Taxonomic Composition

**Figure 6:**
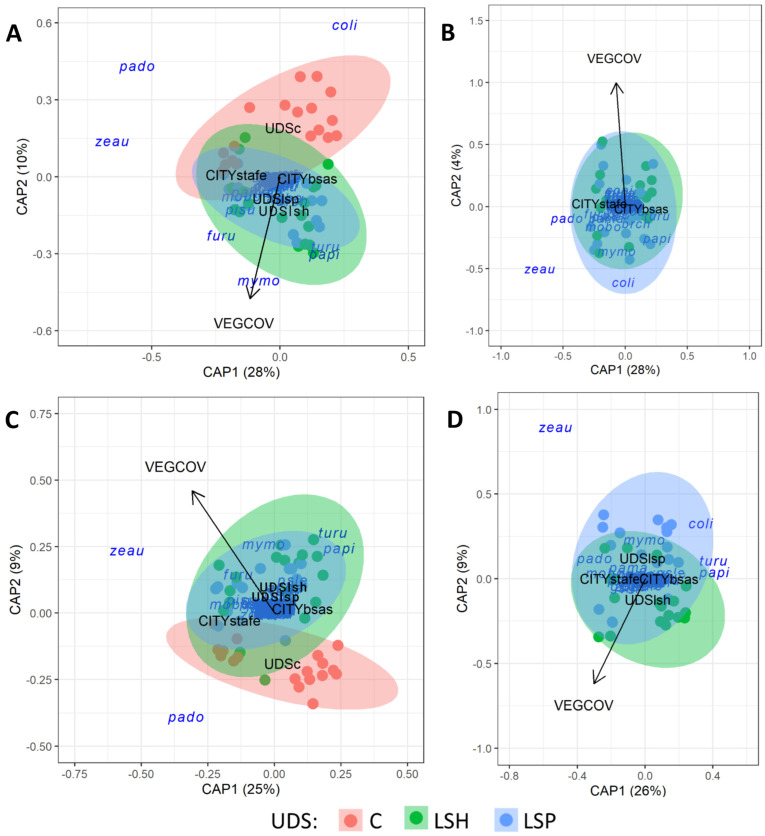
Distance-based redundancy analysis triplots showing the relationship between urban development styles (UDS) (points), species (code), and selected environmental variables (blue arrows) in Santa Fe and Buenos Aires city during the non-breeding (**A**,**B**) and breeding seasons (**C**,**D**). The right column shows analysis without control sites (**B**,**D**). The lines represent the direction (orientation with respect to the axis) and strength (length of the line) of the correlations between environmental variables and variation in species composition. Abbreviations: CITYstafe—Santa Fe city; CITYbsas—Buenos Aires city; VEGCOV—percentage vegetation cover surrounding sites; UDSc—control sites; UDSlsh—land-sharing sites; UDSlsp—land-sparing sites; coli—*Columba livia*; pado—*Passer domesticus*; zeau—*Zenaida auriculata*; mobo—*Molothrus bonariensis*; pisu—*Pitangus sulphuratus*; furu—*Furnarius rufus*; papi—*Patagioenas picazuro*; pama—*Patagioenas maculosa*; mymo—*Myiopsitta monachus*; brch—*Brotogeris chiriri*; psle—*Psittacara leucophthalmus*.

The results of the db-RDA showed that over 40% of the variation in species composition was associated to urban development style, city, and surrounding vegetation coverage during the non-breeding and breeding seasons (non-breeding season: F = 8.5, *p* = 0.001, breeding season: F = 8.4, *p* = 0.001; Figure 6A,C). In ordinations for both the non-breeding and breeding seasons, the first axis showed differences in species composition between cities. The second axis was correlated with surrounding coverage of vegetation and urban development styles. The abundance of *Columba livia* and *Passer domesticus* tended to be higher in control from Buenos Aires and Santa Fe, respectively, and areas with the lowest landscape vegetation coverage (Figure 6A,C). By contrast, the abundance of *Pitangus sulphuratus*, *Furnarius rufus*, *Myiopsitta monachus*, *Turdus rufiventris*, and *Patagioenas picazuro* tended to be higher in land-sharing and land-sparing areas and in areas with the highest surrounding vegetation coverage (Figure 6A,C). 

However, species composition changed between land-sparing and land-sharing landscapes during the breeding season. During the non-breeding season, db-RDA2 only showed significant associations between bird composition and surrounding vegetation coverage and cities (F = 8.1, *p* = 0.001; Figure 6B), but no bird composition differences between land-sparing and land-sharing development styles. During the breeding season, species composition was related to urban development style, cities, and surrounding vegetation coverage (F = 6.6, *p* = 0.001; Figure 6D). The abundance of *Zenaida auriculata*, *Myiopsitta monachus*, *Columba livia*, *Passer domesticus*, and *Turdus rufiventris* was higher in the land-sparing landscape, whereas *Patagioenas picazuro*, *Progne chalybea*, *Furnarius rufus*, *Molothrus bonariensis*, *Pitangus sulphuratus*, *Troglodytes aedon*, *Zonotrichia capensis*, and *Agelaioides badius* were more abundant in the land-sharing landscape (Figure 6D, Table 1). The abundance of *Zenaida auriculata* and *Passer domesticus* was higher in Santa Fe city, whereas the abundance of *Columba livia*, *Turdus rufiventris*, and *Patagioenas picazuro* was higher in Buenos Aires city (Figure 6D, Table 1). Finally, *Columba livia* and *Zenaida auriculata* dominated sites with the lowest surrounding vegetation coverage (Figure 6D). 

## 4. Discussion

Spatial configuration of green cover can affect bird assemblages. Land-sparing and land-sharing development styles supported bird assemblages with different species diversity and composition during the breeding season in the cities of Santa Fe and Buenos Aires. In Buenos Aires, land-sparing favored species richness while land-sharing enhanced the Shannon diversity and the Simpson diversity during the breeding season. In Santa Fe, both urban development styles supported similar species richness and diversity. Thus, our results support that both urban development styles can influence the diversity and composition of birds in Argentinian urban environments. On the other hand, differences in the response of bird assemblages between cities suggest that local knowledge about the effect of urbanization on bird assemblages is required for planning conservation strategies. In addition, pedestrian traffic and the amount of green cover surrounding sites affected bird communities. 

Our study showed that the relationship between urban development style and species richness varied according to cities and seasons. In Buenos Aires, land-sparing had higher species richness than land-sharing during the breeding season. Land-sparing may favor the presence of specialist bird species that require contiguous extensions of green cover [16]. Simpson diversity, which represents the number of dominant species, was higher in land-sharing. Land-sharing has more edge habitats due to the impervious surface interspersed with a wide range of small public and private urban green spaces such as small parks, gardens, and wooded streets. This habitat structure may favor a greater number of dominant species than in land-sparing. In contrast, in Santa Fe city, species diversity had similar values between urban development styles. The lack of association between bird assemblages and the spatial configuration of vegetation was also reported in a previous work performed in another Latin-American city [15]. Our results suggest that the underlying ecological processes that shape bird assemblages in both cities may be different. Further studies are required to provide more conclusive insights about the ecological processes that take place in both cities. Additionally, comparisons with previous studies are difficult because of the differences of methodological approaches, the scale of the study, and the attribute of the bird assemblage analyzed [14,15,16]. In this sense, we followed the methodological approach applied in Ibañez-Álamo et al. [14] and the response of bird assemblages in European cities is different from that of Argentinian cities. Therefore, it is recommended to extend the same methodological approach to different cities to analyze which patterns can be generalizable. 

Distinct urban development styles supported bird assemblages with different species composition. In land-sparing, there was a greater abundance of ground feeder species with a high propensity to form flocks for feeding and breeding such as *Zenaida auriculata* and *Columba livia* [67,68]. These species may require contiguous extensions of lawn for feeding. *Turdus rufiventris*, which feeds on worms and insects on the ground, also was more abundant in land-sparing. In Argentinian cities, high abundances of these species were often reported in large urban parks, which resemble land-sparing development style [69,70,71]. On the other hand, land-sharing can provide a mixture of artificial and natural resources. The highest abundances of species such as *Furnarius rufus*, *Troglodytes aedon*, and *Progne chalybea* may be associated with their ability to nest on artificial structures and feed on the surrounding vegetation or air [69,72,73,74]. Other species more common in land-sharing, such as *Pitangus sulphuratus*, *Molothrus bonariensis*, *Agelaioides badius*, and *Zonotrichia capensis*, were often reported in residential areas along urbanization gradients [69,75,76] and can use artificial structures for nesting (L. M. Leveau pers. obs.). Moreover, additional mechanisms, such as inter-specific interactions, have been associated with changes in species occurrences and abundances between land-sparing and land-sharing development styles in European cities [14,77]. Studies aiming to analyze the ecological processes that shape species composition in urban environments are strongly encouraged due to gaps of knowledge in urban areas of Latin America [78]. 

The relationship between bird communities and urban development styles varied according to seasons. We found differences in species diversity and composition between land-sparing and land-sharing development styles only during the breeding season. Most of the species recorded in both cities can exploit resources present in urban environments [73,79]. As the home range of most of the bird species tends to be larger during the non-breeding season, the availability of resources and not their spatial arrangement in urban environments may influence the presence and abundance of species. However, the spatial arrangement of resources in urban environments may become relevant when species reduce their home range during the breeding season [37,80]. For instance, birds may prefer to nest on sites where they can find resources without getting far from their offspring [37,80]. However, in European cities, differences in the bird assemblage supported by land-sparing and land-sharing development styles were found during the non-breeding season [14]. These patterns highlight the importance of conducting studies during the breeding and non-breeding seasons since the response of bird assemblages to urban development styles may be heterogeneous not only spatially, i.e., between urban development styles, but also temporally [18]. 

Human disturbance is another factor that can influence bird diversity in urban development styles. It can be described in terms of pedestrian traffic, human density, and speed of approaching humans to birds [81,82]. We found a negative relationship between pedestrian traffic and bird diversity [83]. Pedestrian rate can reduce species occupation and persistence and affect the feeding activity of birds [81,84,85]. According to our results, urban planners should take into account strategies for pedestrian traffic calming to enhance species diversity and composition of bird assemblages in any urban development style [34,83]. However, additional mechanisms should be taken into account in further studies. During the survey, we recorded people that occurred within the point count, but we did not record their activity. It has been shown that patterns of human activity, such as bird feeding, can shape the presence and abundance of birds in urban green spaces [86]. Therefore, future studies should shed light on this topic that is still poorly understood. On the other hand, no association pattern was found between species diversity and environmental noise. This result was unexpected since it has been shown that vehicle traffic, which is one of the most frequently cited sources of environmental noise, affected attributes of bird assemblages in both cities [69,83]. However, environmental noise is often associated to changes in urban land use, i.e., urban cores (administrative and commercial areas) usually present higher levels of noise than suburban and peri-urban areas [79]. In our study, changes in bird diversity associated to changes in environmental noise may not be significant since paired samples of urban development styles were located across the urban matrix. In this sense, bird diversity in control was always lower than land-sparing and land-sharing development styles, even if rectangles were located in the urban core or in suburban/peri-urban areas. 

It is often reported that bird diversity is negatively associated to distance to the surrounding natural habitats [87,88]. Natural habitats can act as source habitats, and consequently, the flow of individuals from different species towards the urban landscape can take place. Riparian corridors of the La Plata basin represent highly dynamic and heterogeneous habitats with high levels of biodiversity as a result of an ecotone of species assemblages from tropical and temperate regions [89]. In this sense, Santa Fe and Buenos Aires city are surrounded by these riparian environments, and consequently, we hypothesized a negative distance effect. The lack of association between the distance to the main watercourses and bird diversity may be related to the following ecological processes. First, bird assemblages within the urban matrix of both cities present a subset of native species than in surrounding riparian corridors [90]. It has been shown that urban systems can filter bird species according to their ecological traits [75,91]. In both cities, bird assemblages were composed of species that were often considered as urban exploiters and urban adapters, i.e., species that are able to inhabit and exploit urban habitats [92]. In addition, the success of colonizing urban systems may also be associated with the abundance of species in surrounding non-urban areas [93]. Since knowledge about ecological processes that shape bird assemblages can help to define conservation strategies within cities (e.g., Leveau [94]), future studies aiming to understand the effect of surrounding non-urban habitats on urban bird assemblages are required. 

Bird diversity was influenced by the coverage of vegetation at both the local and landscape level. It has been widely documented that the coverage of vegetation is positively associated to bird diversity since it implies more resources to species [94,95]. Our results agree with these previous findings. However, in Buenos Aires, we found a negative association between the coverage of vegetation and bird diversity at the landscape level. Although these results were unexpected, this negative association may be caused by the low variability in the coverage of vegetation at the landscape scale between sample sites. Most of the landscapes surrounding our sites (>90%) presented a vegetation coverage lower than 40%. Buenos Aires is a compact city where impervious surfaces predominate over vegetation across the urban matrix [96]. In this context, urban green spaces with the highest vegetation coverage within the city may support the highest levels of bird diversity because of a greater availability of resources than surrounding areas [70]. On the other hand, not only the coverage of vegetation is a determinant of resources for birds in urban environments, but also the vegetation diversity and composition [97], which were not assessed in our study. 

The abundance of *Zenaida auriculata*, *Furnarius rufus*, *Myiopsitta monachus*, *Turdus rufiventris*, and *Patagioenas picazuro* was higher in land-sparing/land-sharing urban development styles than in controls and in areas with higher percentage of vegetation coverage at the landscape scale. These species can inhabit cities such as Buenos Aires and Santa Fe, where urban green spaces are characterized by a mixture of non-vegetated infrastructure and vegetation mainly composed of non-native and ornamental species [69,73,83]. By contrast, the abundance of *Columba livia* and *Passer domesticus* increased in control sites with low vegetation coverage at the landscape scale. These species were often considered as urban exploiters in cities worldwide because of generalist habits which allow them to feed on food discarded by humans and nest in buildings [28,69,98].

Land-sparing and land-sharing strategies should be taken into account in future urban planning to enhance biodiversity within the urban matrix in both cities. However, we emphasize three important potential extensions of our work. Firstly, we have considered taxonomic diversity and composition of bird assemblages. Follow-up work needs to examine other attributes of bird assemblages such as functional and phylogenetic diversity and abundance of native and exotic species to reduce gaps of information in the association between urban development style and bird assemblages [14,15,16]. Secondly, it is necessary to analyze the potential synergistic and interactive role that the two urban development styles can play. For example, the inclusion of two urban development styles may have positive ecological impacts by increasing connectivity within the urban matrix. Habitats more typical of a land-sharing development style such as small urban parks and wooded streets were often reported as urban corridors, favoring connectivity between large urban parks and increasing bird diversity [99,100,101]. Thirdly, enhancement of biodiversity in urban ecosystems can be quite important as some evidence suggests that personal exposure to natural features in everyday life is a major determinant of sensitivity to environmental issues [102,103]. Consequently, the third worthwhile extension would examine how the diversity and composition of bird assemblages in each urban development style influence human well-being [104,105,106,107].

Our conceptualization and spatial representation of land-sparing and land-sharing development styles corresponded to urban habitats with structural differences, i.e., differences in spatial configuration of vegetation cover. However, the association pattern between the urban development style and bird assemblages may vary according to land use. For example, the configuration of vegetation cover can be analyzed in different land uses, such as commercial and industrial areas. Previous works have shown differences in the diversity and composition of bird assemblages in urban areas related to differences in land use and/or land cover [32,108]. This is an important extension of research since planning strategies generally reflect decisions based on both land use and land cover.

## 5. Conclusions

We found that the response of bird assemblages to urban development styles varied according to the season and the city. Differences in species diversity between land-sparing and land-sharing were restricted to Buenos Aires city during the breeding season. In contrast, differences in species composition were found in both cities during the breeding season. Due to the fact that both development styles benefit different species, the design of equal proportions of land-sharing and land-sparing landscapes will enhance the total bird diversity in the cities of Santa Fe and Buenos Aires. However, differences in these association patterns with other studies question the extent to which these patterns can be generalizable, and consequently, further studies are required to shed light on the local ecological processes that shape bird assemblages in cities. Additionally, strategies for calming pedestrian traffic and increasing vegetation coverage should be taken into account in future urban planning in order to enhance bird diversity within the urban matrix.

## Figures and Tables

**Table 1:** List of species observed in control, land-sharing, and land-sparing landscapes during breeding (BS) and non-breeding (NBS) seasons in Santa Fe and Buenos Aires, Argentina. The sum of the highest number of individuals recorded during three visits in each urban development style and season is shown for each species. Nomenclature follows the South American Classification Committee of the American Ornithologists’ Union (Remsen et al., 2022).

Scientific Name	Santa Fe City						Buenos Aires City					
	Control		Land-Sharing		Land-Sparing		Control		Land-Sharing		Land-Sparing	
	NBS	BS	NBS	BS	NBS	BS	NBS	BS	NBS	BS	NBS	BS
*Columba livia*	12	5	27	32	4	8	50	92	7	12	31	32
*Patagioenas maculosa*	2	0	3	4	10	5	0	0	0	0	2	0
*Patagioenas picazuro*	0	0	3	2	3	2	3	5	12	10	32	12
*Leptotila verreauxi*	0	0	0	0	0	1	0	0	0	0	0	0
*Zenaida auriculata*	17	24	20	26	26	46	7	12	11	25	17	49
*Columbina picui*	0	1	1	1	16	2	3	2	0	0	0	1
*Guira guira*	8	0	3	8	5	8	0	0	0	0	0	0
*Tapera naevia*	0	0	0	0	0	1	0	0	0	0	0	0
*Chlorostilbon lucidus*	0	3	0	2	0	1	0	1	0	3	0	1
*Hylocharis chrysura*	0	0	1	0	1	1	1	1	2	2	1	1
*Vanellus chilensis*	0	0	2	3	3	2	0	0	0	0	0	0
*Cathartes aura*	0	0	0	0	0	1	0	0	0	0	0	0
*Rostrhamus sociabilis*	0	0	0	0	1	0	0	0	0	0	0	0
*Rupornis magnirostris*	0	0	1	1	1	0	0	0	0	0	0	0
*Parabuteo unicinctus*	0	0	0	0	0	0	0	0	2	3	0	1
*Athene cunicularia*	0	0	2	1	0	0	0	0	0	0	0	0
*Picumnus cirratus*	0	0	0	1	0	0	0	0	0	0	0	0
*Melanerpes cactorum*	0	0	2	1	2	0	0	0	0	0	0	0
*Dryobates mixtus*	0	0	0	1	0	1	0	0	1	0	0	0
*Colaptes melanochloros*	0	0	0	2	3	1	0	0	1	2	2	1
*Colaptes campestris*	0	0	1	1	0	1	0	0	0	0	0	0
*Caracara plancus*	2	0	1	0	2	0	0	0	0	2	2	2
*Falco sparverius*	0	0	1	2	0	1	0	0	0	0	0	0
*Myiopsitta monachus*	4	2	15	7	11	17	2	0	24	7	22	12
*Brotogeris chiriri*	0	0	0	0	0	0	0	0	2	4	21	6
*Amazona aestiva*	0	0	0	0	0	0	0	0	0	2	3	2
*Pyrrhura frontalis*	0	0	0	0	0	0	6	1	4	14	12	5
*Pyrrhura molinae*	0	0	0	0	0	0	0	0	0	0	0	2
*Aratinga nenday*	0	0	0	0	0	0	0	0	0	5	5	4
*Psittacara leucophthalmus*	0	0	0	0	0	0	0	2	0	11	0	8
*Taraba major*	0	0	0	0	1	0	0	0	0	0	0	0
*Lepidocolaptes angustirostris*	0	0	2	2	1	1	0	0	2	1	1	1
*Furnarius rufus*	5	5	6	5	12	8	2	2	5	9	5	4
*Phacellodomus ruber*	0	0	0	0	2	2	0	0	0	0	0	0
*Pseudoseisura lophotes*	0	0	3	1	3	2	0	0	0	0	0	0
*Schoeniophylax phryganophilus*	0	0	2	0	2	1	0	0	0	0	0	0
*Camptostoma obsoletum*	0	0	1	1	1	1	0	0	0	0	0	0
*Serpophaga subcristata*	0	0	0	0	0	0	0	0	1	2	1	1
*Serpophaga griseicapilla*	0	0	1	0	0	0	0	0	0	0	0	0
*Pitangus sulphuratus*	3	5	4	6	4	9	3	3	4	5	3	3
*Machetornis rixosa*	1	1	2	2	1	3	0	0	1	3	1	4
*Myiodynastes maculatus*	0	0	0	0	0	0	0	0	0	0	0	1
*Tyrannus melancholicus*	0	0	0	1	0	2	0	0	0	1	0	0
*Tyrannus savana*	0	0	0	2	0	3	0	0	0	1	0	1
*Sublegatus modestus*	0	0	0	1	0	0	0	0	0	0	0	0
*Cyclarhis gujanensis*	0	1	1	1	1	1	0	0	0	0	0	0
*Progne tapera*	0	0	0	5	0	4	0	0	0	1	0	1
*Progne chalybea*	0	4	0	6	0	7	0	5	0	6	0	4
*Tachycineta leucorrhoa*	0	1	3	2	0	4	0	1	5	5	0	3
*Troglodytes aedon*	1	3	3	3	2	2	2	2	3	4	2	4
*Polioptila dumicola*	0	0	3	2	2	2	0	0	0	0	0	1
*Turdus rufiventris*	1	0	2	2	2	2	3	4	11	7	12	8
*Turdus amaurochalinus*	0	0	5	1	2	1	0	0	0	1	0	1
*Mimus saturninus*	4	2	5	2	3	0	0	1	4	2	2	3
*Mimus triurus*	0	0	1	0	0	0	0	0	0	0	0	1
*Sturnus vulgaris*	0	0	4	2	0	2	0	1	0	3	7	16
*Passer domesticus*	13	18	15	9	11	12	6	5	2	5	12	13
*Spinus magellanicus*	0	1	0	0	2	2	0	0	0	3	2	1
*Zonotrichia capensis*	2	2	3	2	2	3	1	1	1	2	1	1
*Icterus pyrrhopterus*	0	0	2	0	0	0	0	0	0	1	0	1
*Molothrus rufoaxillaris*	0	0	0	0	1	1	0	0	1	2	0	3
*Molothrus bonariensis*	5	7	5	5	21	15	1	0	1	4	12	1
*Agelaioides badius*	0	0	4	4	4	0	0	0	7	3	7	4
*Geothlypis aequinoctialis*	0	0	0	0	0	1	0	0	0	0	0	0
*Setophaga pitiayumi*	0	0	0	1	0	1	0	0	1	1	1	1
*Piranga flava*	0	0	0	0	0	1	0	0	1	2	0	0
*Sicalis flaveola*	0	1	1	3	3	2	0	0	2	1	0	2
*Sicalis luteola*	0	0	0	0	0	2	0	0	0	0	0	0
*Saltator coerulescens*	0	0	0	0	0	1	0	0	0	0	0	0
*Paroaria coronata*	0	2	0	4	2	3	0	0	0	0	0	1
*Paroaria capitata*	0	0	0	1	0	1	0	0	0	0	0	0
*Thraupis sayaca*	0	1	1	2	2	2	2	2	1	2	0	2

**Table 2:** Final generalized linear models between bird diversity and environmental variables during (a) non-breeding and (b) breeding seasons: indicates interaction between variables.

Response Variable	Predictor	Estimate	Standard Error	z Test/ *t*-test	*p*
(a) Non-breeding season					
Species richness	Intercept	3.23	0.23	13.6	<0.001
	Landscape_control	−0.69	0.12	−5.66	<0.001
	Landscape_land-sharing	−0.16	0.1	−1.63	0.1
	Pedestrians	−0.01	0.004	−3.77	<0.001
	Vegetation	−0.01	0.01	−2.26	0.02
	City_SantaFe	−0.24	0.2	−1.17	0.24
	Vegetation:City_SantaFe	0.01	0.01	2.23	0.026
Shannon diversity	Intercept	12.39	1.55	7.97	<0.001
	Landscape_control	−4.29	0.79	−5.4	<0.001
	Landscape_land-sharing	−0.6	0.75	−0.81	0.42
	Pedestrians	−0.09	0.03	−3.55	<0.001
	Vegetation	−0.07	0.04	−1.87	0.07
	City_SantaFe	−2.19	1.3	−1.68	0.1
	Vegetation:City_SantaFe	0.11	0.04	2.66	0.01
Simpson diversity	Intercept	8.32	0.59	14.07	<0.001
	Landscape_control	−3.03	0.62	−4.93	<0.001
	Landscape_land-sharing	−0.29	0.63	−0.47	0.64
	Pedestrians	−0.06	0.02	−4.17	<0.001
(b) Breeding season					
Species richness	Intercept	3.17	0.07	44.97	<0.001
	Landscape_control	−0.75	0.09	−7.92	<0.001
	Landscape_land-sharing	−5	0.07	−0.66	0.51
	Pedestrians	−0.01	0.003	−3.24	0.001
Shannon diversity	Intercept	8.63	1.36	6.36	<0.001
	Landscape_control	−2.21	1.52	−1.45	0.15
	Landscape_land-sharing	5.25	1.87	2.8	0.007
	Vegetation	0.04	0.05	0.9	0.37
	City_SantaFe	−2.02	1.36	−1.48	0.14
	Landscape_control:Vegetation	−0.13	0.06	−2.07	0.04
	Landscape_land-sharing:Vegetation	−0.09	0.06	−1.6	0.11
	Vegetation:City_SantaFe	0.13	0.05	2.67	0.01
Simpson diversity	Intercept	6.84	1.02	6.69	<0.001
	Landscape_control	−2.71	0.79	−3.42	0.001
	Landscape_land-sharing	2.82	0.71	3.99	<0.001
	Vegetation	−0.002	0.03	−0.05	0.96
	City_SantaFe	−2.35	1.11	−2.12	0.04
	Vegetation:City_SantaFe	0.1	0.04	2.67	0.01

## Data Availability

The datasets generated and/or analyzed during the current study are available upon request to the corresponding author.

## Supplementary Materials

The following supporting information can be downloaded at: https://www.mdpi.com/article/10.3390/ani13050894/s1, Figure S1: Image processing for the classification of sampling units as an urban development style; Figure S2: Ordination of sampling units from the city of Santa Fe and Buenos Aires obtained from a Principal Component Analysis; Figure S3: Sample completeness curves for each urban development style in the city of Santa Fe and Buenos Aires; Figure S4: Diagnostics of fitted generalized linear models for all response variables.
