# Supplementary figures and images for: Responses of Urban Bird Assemblages to Land-Sparing and Land-Sharing Development Styles in Two Argentinian Cities
Source: Animals (Basel). 2023 Mar 1;13(5):894. doi: 10.3390/ani13050894 (PMC10000187; doi:10.3390/ani13050894)

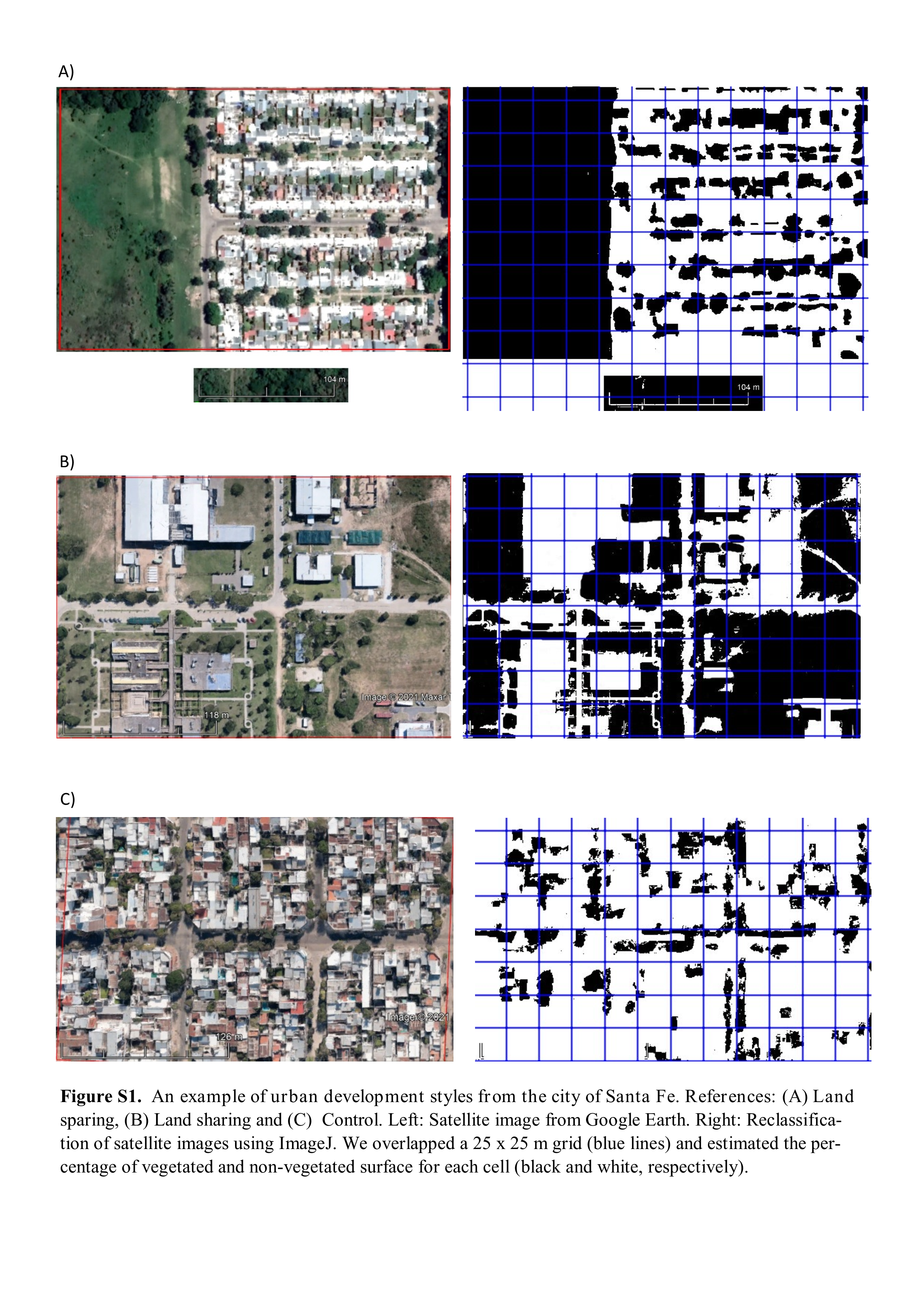

Supplement: Supplementary file 1 [file animals-13-00894-s001.zip › Figure S1 Urban development style_lsplshc.tif]

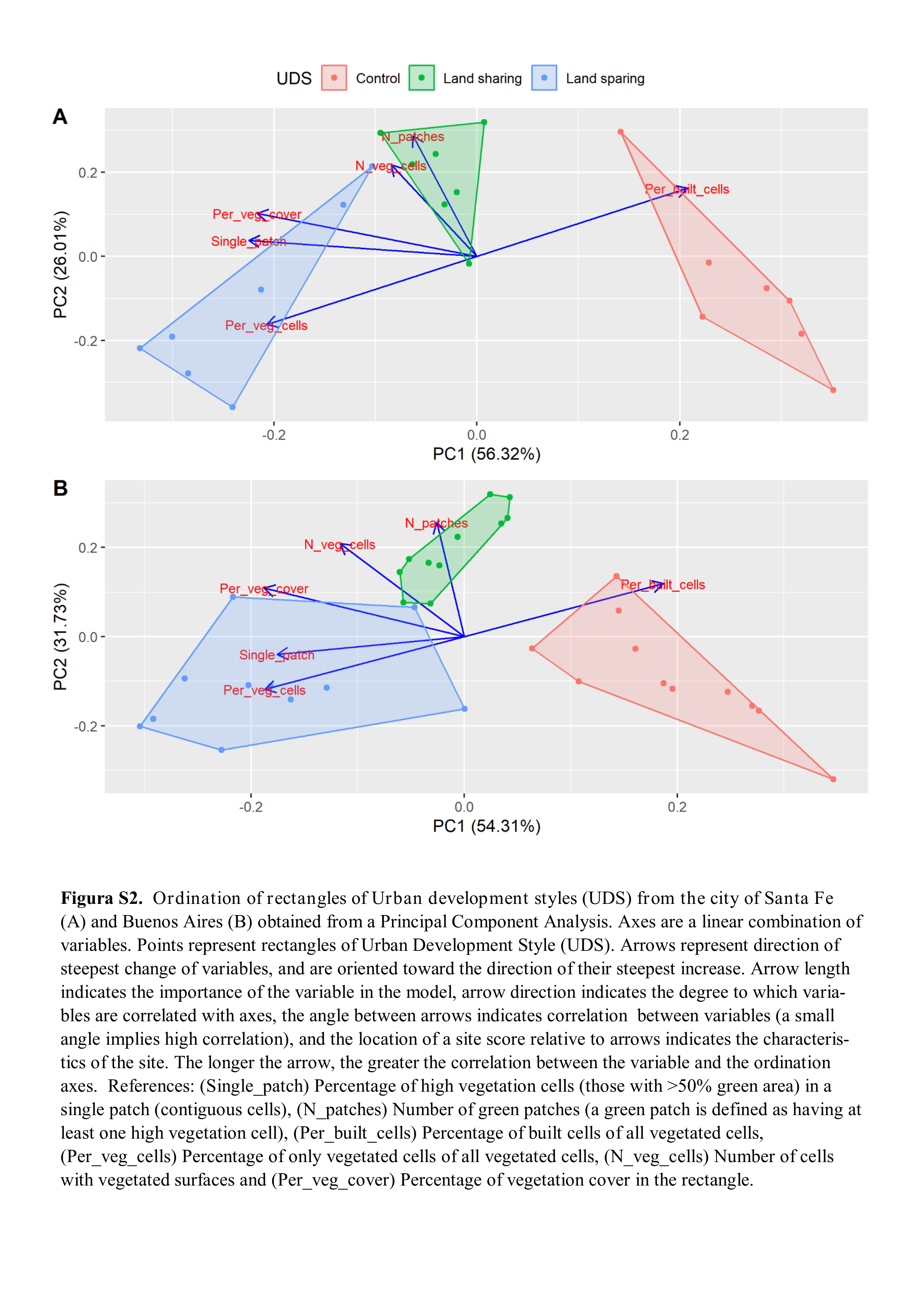

Supplement: Supplementary file 1 [file animals-13-00894-s001.zip › Figure S2 PCA_RECTANGLES_UDS_STAFE_BS.tif]

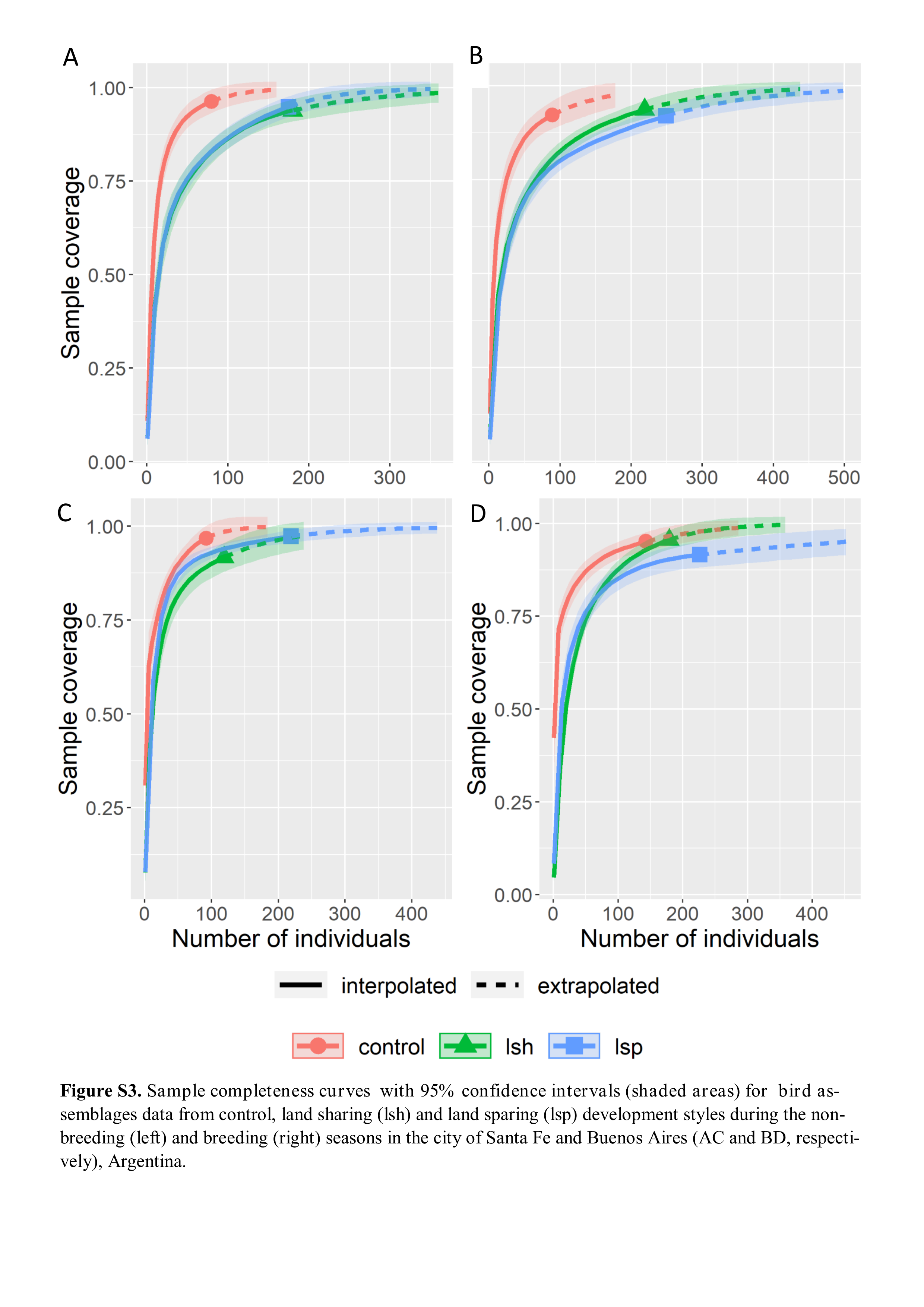

Supplement: Supplementary file 1 [file animals-13-00894-s001.zip › Figure S3 Sample_completeness.tif]

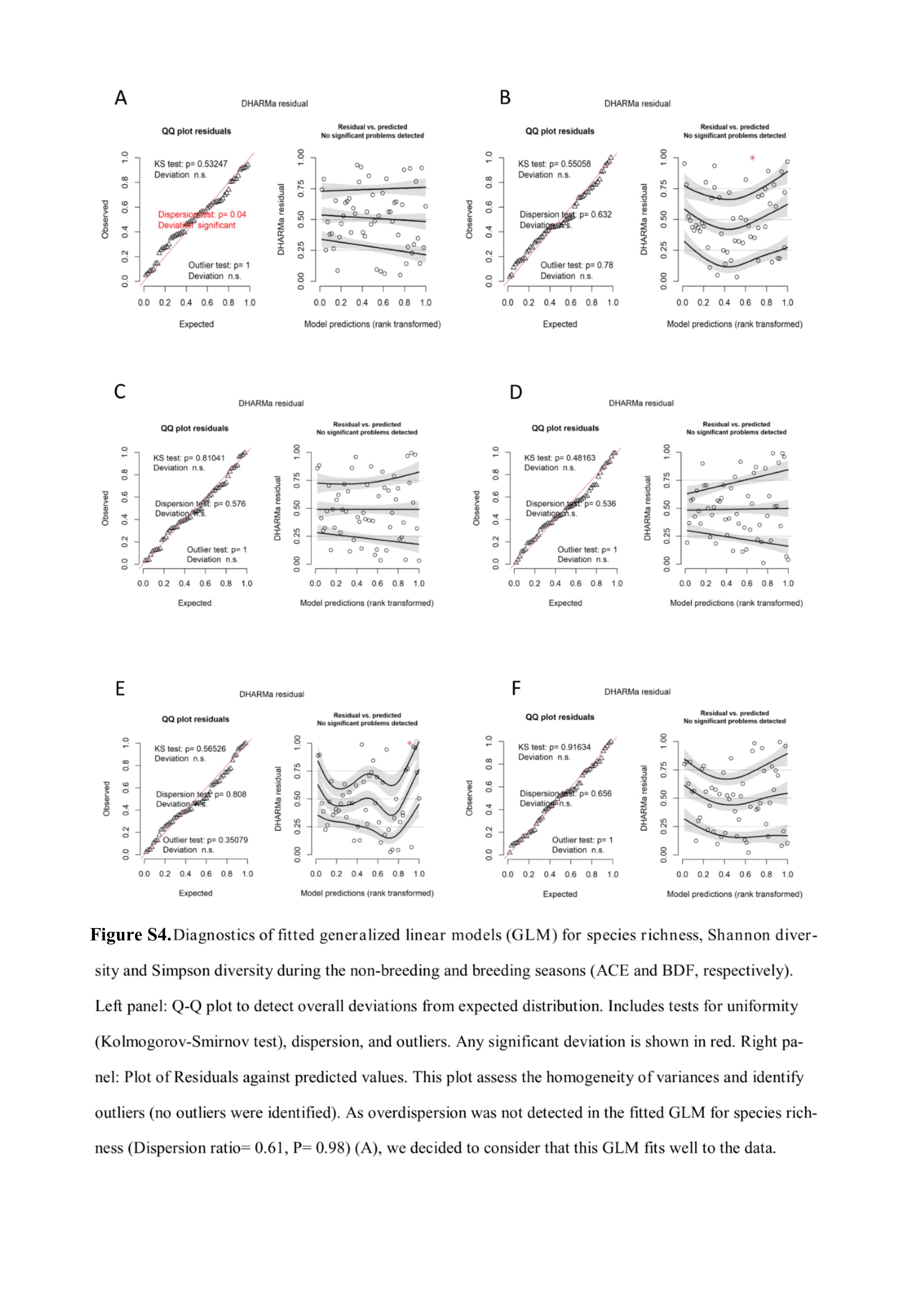

Supplement: Supplementary file 1 [file animals-13-00894-s001.zip › Figure S4 Diagnostic_GLM_V2.tif]
